# A Prospective Approach to Integration of AI Fracture Detection Software in Radiographs into Clinical Workflow

**DOI:** 10.3390/life13010223

**Published:** 2023-01-13

**Authors:** Jonas Oppenheimer, Sophia Lüken, Bernd Hamm, Stefan Markus Niehues

**Affiliations:** 1 Klinik für Radiologie, Charité Universitätsmedizin Berlin, Hindenburgdamm 30, 12203 Berlin, Germany; 2 Berlin Institute of Health, Anna-Louisa-Karsch-Straße 2, 10178 Berlin, Germany

**Keywords:** artificial intelligence, radiographs, fracture, computer-aided diagnosis

## Abstract

Gleamer BoneView^©^ is a commercially available AI algorithm for fracture detection in radiographs. We aim to test if the algorithm can assist in better sensitivity and specificity for fracture detection by residents with prospective integration into clinical workflow. Radiographs with inquiry for fracture initially reviewed by two residents were randomly assigned and included. A preliminary diagnosis of a possible fracture was made. Thereafter, the AI decision on presence and location of possible fractures was shown and changes to diagnosis could be made. Final diagnosis of fracture was made by a board-certified radiologist with over eight years of experience, or if available, cross-sectional imaging. Sensitivity and specificity of the human report, AI diagnosis, and assisted report were calculated in comparison to the final expert diagnosis. 1163 exams in 735 patients were included, with a total of 367 fractures (31.56%). Pure human sensitivity was 84.74%, and AI sensitivity was 86.92%. Thirty-five changes were made after showing AI results, 33 of which resulted in the correct diagnosis, resulting in 25 additionally found fractures. This resulted in a sensitivity of 91.28% for the assisted report. Specificity was 97.11, 84.67, and 97.36%, respectively. AI assistance showed an increase in sensitivity for both residents, without a loss of specificity.

## 1. Introduction

In recent years, multiple artificial intelligence (AI) diagnostic tools to assist radiologists have become available for purchase after regulatory approval [1,2]. Many of these products apply for approval via retrospective evidence, showing the diagnostic performance on a selected set of data [3]. With most training and testing of the algorithms happening retrospectively, the applicability and use case in the real-life clinical setting often remains vague and unproven. Many AI diagnostic tools only have regulatory approval to assist the radiologist in detection and prioritization, not allowing for a stand-alone diagnosis [3]. 

One such AI tool for fracture detection in radiographs is Gleamer BoneView^©^ (Gleamer, Paris, France), which has shown promising evidence for fracture detection with an accuracy of up to 0.97 area under the curve (AUC) of the AI alone [4,5]. Generally, sensitivity and specificity in fracture detection can vary widely between anatomical regions and experience. In a study of extremity fractures in an emergency department, Wei et al. found 115 missed fractures in a total of 3081 fractures, only one-third of which were radiographically imperceptible [6]. Other research has found up to 3.1% of missed fractures at initial presentation to the emergency department, 86% of which resulted in changes in treatment [7]. Additionally, radiologists can be prone to common diagnostic errors, such as satisfaction of search, and may therefore miss multiple present fractures [8]. Novices miss subtle fractures significantly more often than experts [9]. Missed diagnosis of fractures represents the second most common cause of malpractice suits against radiologists in the United States due to delays in treatment or even the absence thereof, as well as additional radiation exposure with further imaging [10]. These aspects promise to be mitigated by AI software assisting in detecting fractures.

However, due to regulatory hurdles and performance limitations, standalone AI solutions, in which the software, not a radiologist, makes the final diagnostic decision are not currently available on the market [1,3]. While in the future, such solutions may promise better diagnostic statistics than humans while simultaneously cutting costs and the need for personnel resources, a risk of information loss with this approach remains. Current AI solutions, such as Gleamer BoneView^©^ specialize only in one task, such as fracture detection in the given case. If the software is implemented in a standalone setting and radiographs are not reviewed by a human reader, ancillary information may get lost. Currently, the software only proposes a diagnosis of fracture or no fracture, leaving out vital information regularly included in reports, such as level of displacement, fracture shape, and joint involvement. Other critical secondary findings, such as underlying bone disease or even an incidental finding of a chest lesion in a rib radiograph, would also not be conveyed. Until solutions exist that combine the high diagnostic accuracy with secondary findings and a pertinent report for the attending physician, a combination of AI and human diagnosis may be useful for increasing diagnostic accuracy in fracture detection.

AI algorithms are trained on large sets of data, usually previously labeled by experts. Gleamer BoneView^©^ was developed using a set of 60,170 trauma radiographs acquired from 22 institutions. The deep learning algorithm is based on the “Detectron 2” framework [4].

In this prospective study, we propose to test the prospective integration of Gleamer BoneView^©^ into the standard clinical workflow of resident radiologists in a level 3 trauma center to prove if the assistance of AI can lead to better initial reports for radiographs with an inquiry for trauma.

## 2. Materials and Methods

### 2.1. Integration into Clinical Workflow

Regular clinical workflow precludes the patient examination by a clinician in either an outpatient (emergency department (ED) or outpatient clinic) or inpatient setting. If a traumatic or nontraumatic fracture is suspected after the attainment of a patient history and clinical exam, the clinician may order a conventional radiographic exam of any anatomic regions implicated, purveying crucial clinical information to the radiographers and radiologists. These exams are performed by set standards by the radiography staff. Afterwards, the images can directly be viewed in the clinic’s PACS System (Phönix PACS MERLIN Diagnostic Workcenter Version 7.0, Phönix-PACS GmbH, Freiburg, Germany). A resident radiologist immediately issues a preliminary written report (marked as such). All radiographs are finally supervised and reported on within 24 h. These preliminary reports are reviewed and either signed off unchanged or corrected accordingly by an experienced board-certified radiologist. Any pertinent clinical changes from the preliminary to the final report are submitted to the providing clinician where possible.

**Figure 1:**
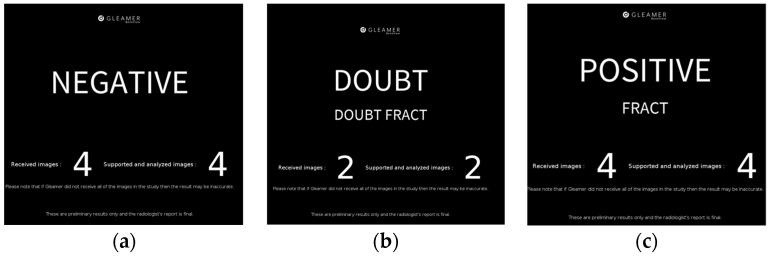
Result frames shown by the AI software delineating a case of exams without a fracture ((**a**), **left**), a possible fracture at 50–89% certainty threshold ((**b**), **center**) and a fracture at ≥90% certainty ((**c**), **right**). The number X-ray images analyzed in the exam is also shown.

**Figure 2:**
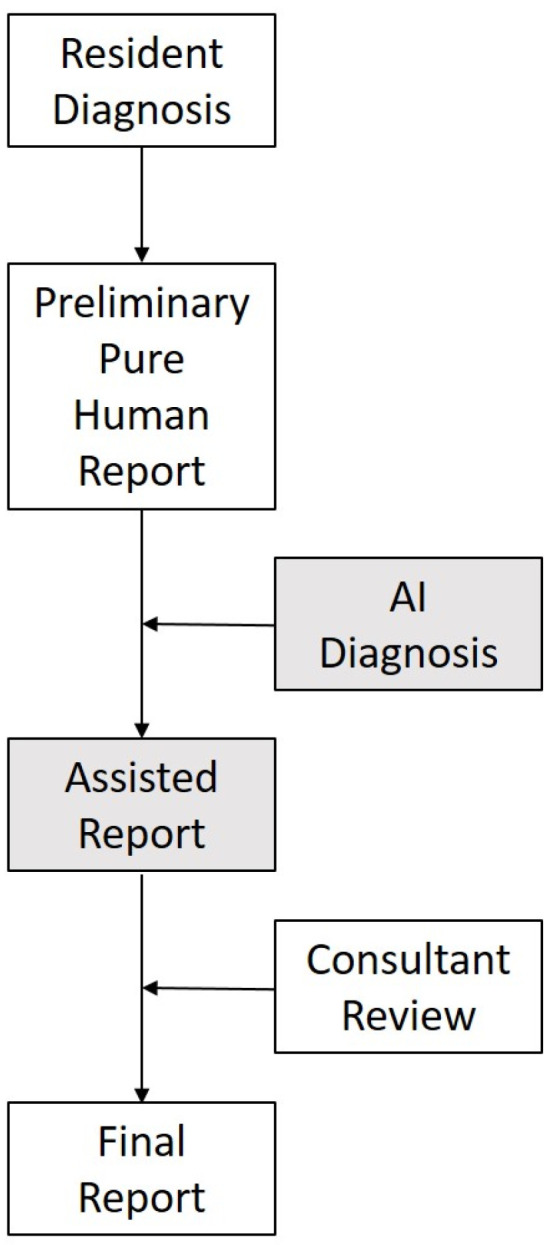
Clinical workflow for fracture diagnosis without (white boxes) and with (gray boxes) AI assistance.

The AI-integrated clinical workflow changes the radiologic reporting slightly to potentially change the fracture diagnosis in the preliminary reports. After making an initial diagnosis on only the images and noting this down, all images of the radiographic exam were transmitted to an onsite interface running the AI fracture detection software (Gleamer BoneView^©^ Version 1.2.0, steady-state version). This software returned a diagnosis of “Positive,” “Doubt,” or “Negative” (Figure 1a–c) for the diagnosis of fractures for the entire exam within 3 min, marking a bounding box with a through line where a fracture is diagnosed and a bounding box with a dashed line where a fracture is possible (“Doubt”) for all possible fractures detected in each image. The AI sets the threshold of “Doubt” at 50–89% confidence and “fracture” at greater than or equal to 90% [4]. Additionally, the AI marks regions of interest where it diagnoses a joint effusion or dislocation. With this AI diagnosis, the resident was able to reconsider their initial diagnosis and then write the preliminary report, after which workflow remained unchanged. For purposes of this study, the resident evaluated the presence or absence of a fracture for all images of the entire acquired exam in a “present”/”absent” manner, regardless of if possibly multiple fractures were visible in one exam. The full result of the AI software, as presented in a separate PACS image was noted, as well as this resulting in any change of the initial diagnosis. For the calculation of AI-only diagnostic measures, “Positive” and “Doubt” were both counted as fracture-positive. This combined result was then noted as “fracture”/”no fracture,” corresponding to the written preliminary report. Finally, once a final report was available, results were compared to the final diagnosis by the board-certified radiologist. Figure 2 shows the clinical workflow with and without AI assistance.

In cases where cross-sectional imaging (CT, MRI, PET) was performed within one week after the initial radiographic exam, and no new trauma or symptoms were indicated, this diagnosis of this imaging was used as the gold-standard reference, noting where the final report diagnosis was overruled.

### 2.2. Inclusion and Exclusion

All radiographic exams with inquiry for fracture, which were primarily reported on by either of two radiology residents (J.O., S.L.) in a total period of five months (February–June 2022) were included in this study. An exam was defined as a single or set of images of the same anatomic region for a single patient, where a fracture was suspected. Each exam is saved as a separate entry in our clinic’s RIS-System (GE Centricity RIS-I 7.0; GE Healthcare, Chicago, IL, USA). Dependent on the type of trauma, multiple exams may be processed for the same patient. 

Inclusion Criteria:

- Full radiographic exam of one anatomic region with one or multiple images;
- Inquiry for fracture;
- Primary review by either of two residents.
- Exclusion Criteria:
- Follow-up imaging for known fractures;
- Skeletal radiographs with other inquiry (i.e., inflammatory disease, post-surgical radiographs, etc.);
- Non-processable radiographs: full chest radiographs, abdomen radiographs, cervical spine radiographs, radiographs of the skull or face.

Patient gender and age were noted for each exam, as well as the number of acquired images. The anatomical region was broadly classified into one of 8 regions (spine, ribs, shoulder/clavicle, elbow/arm, hand/wrist, hip/pelvis, knee/leg, ankle/foot), as used in a previous study on the AI software [4]. In spine radiographs, a further subdivision into thoracic and lumbar spine was made. The imaged side was noted where applicable. The type of trauma leading to the radiograph was also broadly classified into 5 groups: (1) no direct trauma in the patient history, (2) falls, (3) blunt-force trauma (e.g., assault, collision or crush injuries), (4) sharp force trauma (e.g., cuts and bites), (5) trauma due to unnatural joint movement (e.g., supination trauma). In cases with a fracture, an additional classification into obvious (multi-fragmented and/or displaced) and nonobvious (single fracture line and/or nondisplaced) was made. Finally, it was noted if any foreign material was previously present in the imaged bone, broadly classifying this into metal hardware, cement, or a combination of both. 

### 2.3. Statistical Analysis

Data entry and analysis was performed with Excel 365 Version 2208 (Microsoft Corporation, Redmon, WA, USA) and IBM SPSS Statistics 25 (IBM, Armonk, NY, USA). Sensitivity, specificity, positive predictive value (PPV), and negative predictive value (NPV) were calculated for each resident’s cases for human diagnosis, AI-only diagnosis and combined report, compared to the gold standard set by the board-certified radiologist or cross-sectional imaging. A repeated measures ANOVA was used to test statistical significance in the difference of the sensitivity between the three groups. Significance was defined at a *p*-value < 0.05. Additionally, these statistics are reported for the full data set, without subdividing between the residents. Further analysis was performed for each of the 8 defined body regions, as well as comparing between cases with and without foreign materials and obvious to nonobvious fractures and the results for patients under the age of 18. All results are shown with 95% confidence intervals (95%-CI).

Based on an initial dataset with a null hypothesis for the human sensitivity of 78.7% with a 30% prevalence of fractures, the sample size needed for a power greater than 0.8 at a 0.05 significance level in a 2-sided test for an alternative hypothesis of 85% for the combined sensitivity was calculated in 1006 exams. 

## 3. Results

### 3.1. Dataset

A total of 1163 exams (641 for reviewer 1, 522 for reviewer 2) in 735 patients (58% female) were included in the study. Average patient age was 61.39 years (standard deviation 21.9 years, range: 2–100 years). The dataset included at total of 2256 conventional X-ray images (average 1.94 per exam). Hip (225) and spine (219) exams were most common. A total of 367 true positive fractures were defined (31.56%). Additional imaging was available for confirmation of diagnosis in 149 cases (109 CT scans, 33 MRI, 4 CT and MRI, and 3 additional conventional scans). Additional imaging overruled the final report diagnosis in nine cases (seven fractures identified, two fractures ruled out). This results in an expert diagnosis sensitivity of 98.37% and specificity of 99.75%. One hundred and eighty-one fractures were defined as obvious. The AI software rejected the images for analysis in 11 cases (10 spine images, 1 rib images) as being of an unsupported anatomical region (either as constituting a chest radiograph or an abdominal radiograph). These cases were not excluded for further analysis. Only one fracture was present in these eleven cases.

Of the exams, 610 cases were classified as falls, 368 had no apparent trauma history, 105 were blunt force trauma, 32 were sharp force trauma and 48 were classified as distortion injuries.

### 3.2. General Sensitivity and Specificity

Overall sensitivity for humans was 84.79%, for AI alone 86.69%, and combined 91.28%. A repeated measures ANOVA showed statistical significance between the three groups (*p* < 0.001). Specificity was 97.11%, 84.67%, and 97.36% respectively. Table 1 shows the results for the entire dataset as well as each subset of the two reviewers. With the addition of AI software, 35 changes to the initial diagnosis were made in the dataset (3.01%). This resulted in 25 additional correct fracture diagnoses, 6 correct fracture exclusions, as well as 2 incorrect fracture diagnoses and exclusions each.

**Figure 3:**
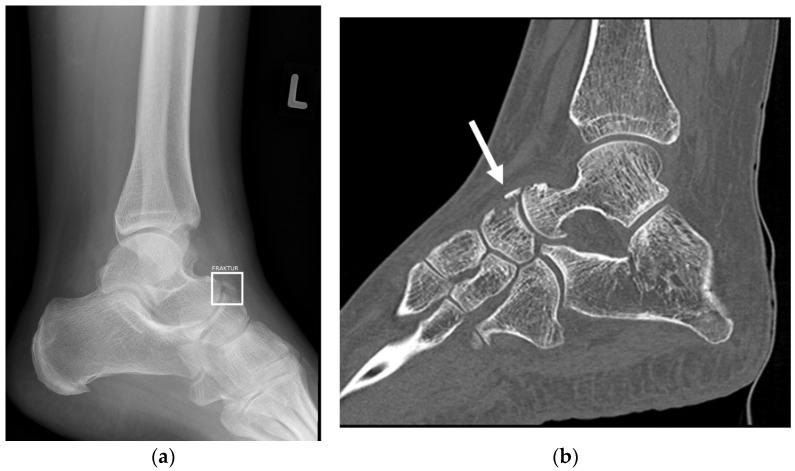
(**a**) shows a lateral radiograph of the left ankle. The bounding box (white) marks a fracture noted by the AI software. This was initially thought to be an osteophyte by both the resident and the board-certified radiologist. (**b**) Sagittal CT imaging confirms a true positive fracture of the Navicular (white arrow).

**Figure 4:**
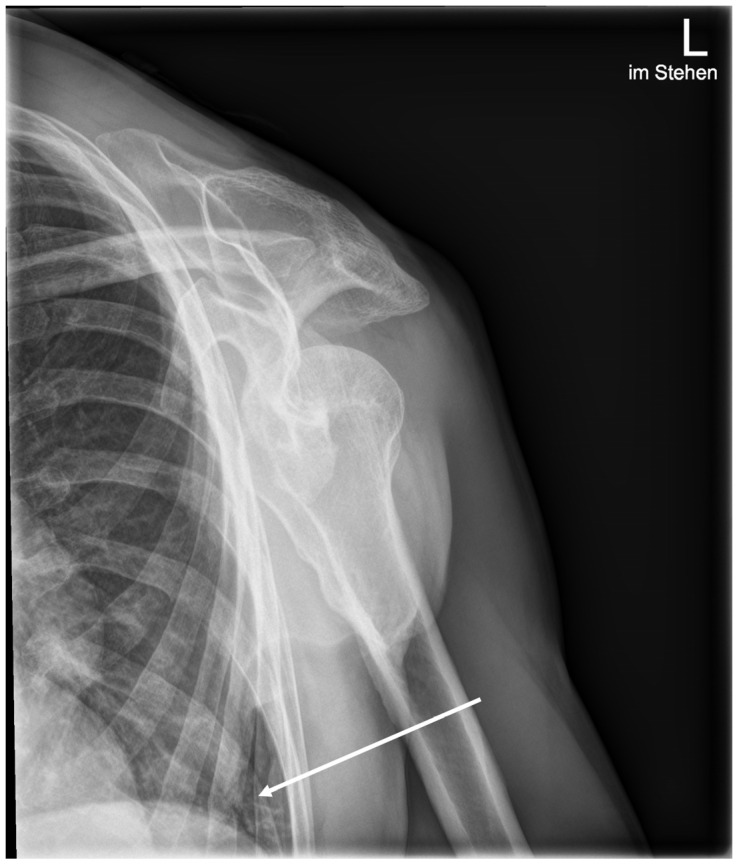
Standing ap radiograph of the left shoulder. The white arrow marks an incidentally caught rib fracture, which was correctly diagnosed by the resident but missed by the AI software.

Figure 3 shows an example of a fracture noted by the AI, which was initially thought to be an osteophyte by both the resident and in the final report. Additional CT imaging performed confirmed the presence of a fracture. A rib fracture incidentally included on a shoulder radiograph was missed by the AI, but correctly diagnosed by the resident (Figure 4).

### 3.3. Sensitivity and Specificity by Anatomical Region

Highest sensitivity for the human reader was for hip/pelvis exams (93.33%), lowest for ribcage images (64.29%). Specificity was above 90% for all regions. AI sensitivity was highest in shoulder/clavicle images (91.11%) and only fell below 80% in ribcage exams. Specificity varied widely between 62.20% in spine radiographs and 93.98% for knee/leg exams. Combined sensitivity was above 80% in all regions except ribcage (78.57%). Specificity was above 90% for all regions and 100% for spine radiographs. Full results for sensitivity and specificity are shown in Table 2.

### 3.4. Foreign Material

A total of 147 cases included foreign material in the images, 94 cases with metal osteosynthesis material, 13 with cement, 35 with cement and metal, 6 cases had casts on, and 1 case had a cast and metal material. Human-only sensitivity in cases with foreign material was above the overall sensitivity with 93.55% (±0.06), specificity was 98.82% (±0.02). AI performance was slightly below the overall results at 82.25% sensitivity (±0.10) and specificity fell to 69.41% (±0.10). Combined results were in line with the overall data set at 93.55% (±0.06) sensitivity and 97.65% (±0.02) specificity. 

### 3.5. Obvious and Nonobvious Fracture

**Figure 5:**
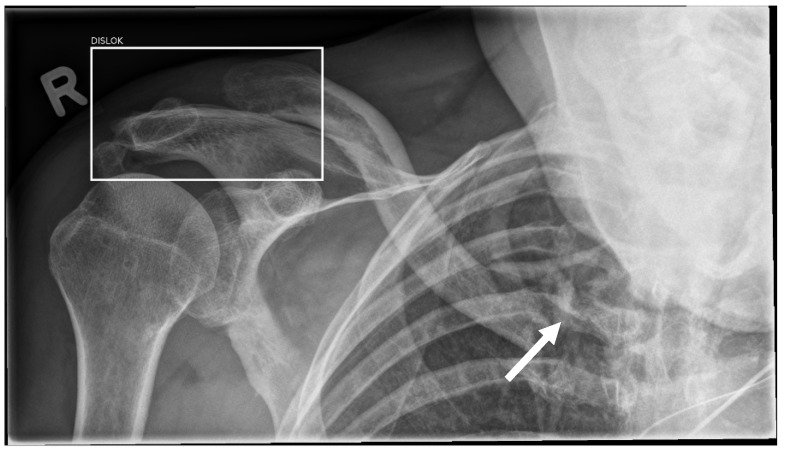
Standing ap radiograph of the right shoulder. The AI software correctly sets a bounding box around the acromioclavicular joint injury (marked as “Dislocation”); however, both the AI and the resident missed the displaced proximal clavicle fracture (white arrow).

Of 181 obvious fractures, three fractures classified as obvious were missed by the human reader, five were missed by the AI. Of these, only one obviously dislocated fracture of the proximal clavicle in a shoulder radiograph was missed by both human and AI, shown in Figure 5. This resulted in a sensitivity of 98.34% for the human reader, 97.23% for AI and 99.44% for the combined diagnosis. One hundred and eighty-six fractures were classified as nonobvious. Sensitivity for the human reader was 71.50%, for AI 76.88% and combined 83.33%.

### 3.6. Fractures in Children

Thirty-one exams were of patients under the age of 18 years, with an average age of 10.8 years. Six of these exams were positive for fractures. Sensitivity for the human reader was 100%, for AI 83.33%, and combined 100%. Specificity was 92.00%, 84.00%, and 92.00%, respectively.

### 3.7. Effusion and Dislocation

Gleamer BoneView^©^ also marks joint effusion and dislocation as possible pathologies in the report. In our dataset, 61 joint dislocations were marked, of which the software correctly marked 32 (52.46%). Forty-six cases were incorrectly marked as dislocated. Twenty-nine cases were reported as having a joint effusion (only noted in elbow, wrist, knee, and ankle radiographs), 21 of which were identified by the software. Thirty cases were incorrectly marked as having an effusion by the AI. This included one shoulder/clavicle radiograph and one hip/pelvis exam, anatomical regions not usually associated with effusion aiding in (indirect) fracture diagnosis.

## 4. Discussion

With the assistance of AI software, resident radiologists were able to significantly improve their sensitivity for fracture detection in radiographs in a prospective setting with integration into clinical workflow. Our results show an increase in sensitivity of 6.54% and are in line with a previous retrospective study that used Gleamer BoneView^©^, in which Guermazi et al. found an increase of 7.6% in sensitivity although also 2.8% in specificity (0.25% in our study) [4]. A retrospective study from Duron et al. also found an increase of 8.7% and 4.1%, respectively [5]. The prospective setting may lead to a slight decrease in the effectiveness of AI assistance, as the gold standard was set by regular clinical practice (expert diagnosis, cross-sectional imaging where necessary) and not by a 3-person consensus decision as in the retrospective studies, but our research more accurately reflects clinical practice. Standalone AI performance was noted at 88% by Guermazi et al., directly in line with our results. A separate study on pediatric patients (2–21 years of age) found a per patient sensitivity of 91.3% with a specificity of 90%, with a low sensitivity of the AI for avulsion fractures [11]. Nguyen et al. were able to find an increase in sensitivity in a retrospective dataset of pediatric patients for fracture detection (73.3% without AI, 82.8% with assistance), with an increase of 10.3% for junior radiologists and 8.2% for senior radiologists [12]. In our study a small subset of pediatric patients is included, representing the patients that presented to our institution’s emergency department.

Various other AI algorithms exist for fracture detection in research as well as commercial settings. In a recent meta-analysis by Kuo et al. 37 studies with AI tools for fracture detection in radiographs were analyzed, reaching a pooled sensitivity of up to 92% and a sensitivity of 91%. However, many of these algorithms were only for one specific anatomic region [13]. For distal radial fractures, sensitivities of 86% and 94% were reached [14,15] while AI was able to detect hip fractures with up to 100% sensitivity and 99% specificity [16]. 

The residents using the AI tool noted that in general they felt an increase of confidence in their diagnosis if AI was concordant with their diagnosis. In the cases where the diagnoses diverged, the AI diagnosis helped in a small number “obvious” misses, highlighting the role as a second viewer when satisfaction of search, tiredness or distraction can cause the radiologist to make a false call. However, in some edge cases, both residents reported feeling somewhat unsure of their diagnosis, in particular if they decided on a fracture and the AI result was negative.

**Figure 6:**
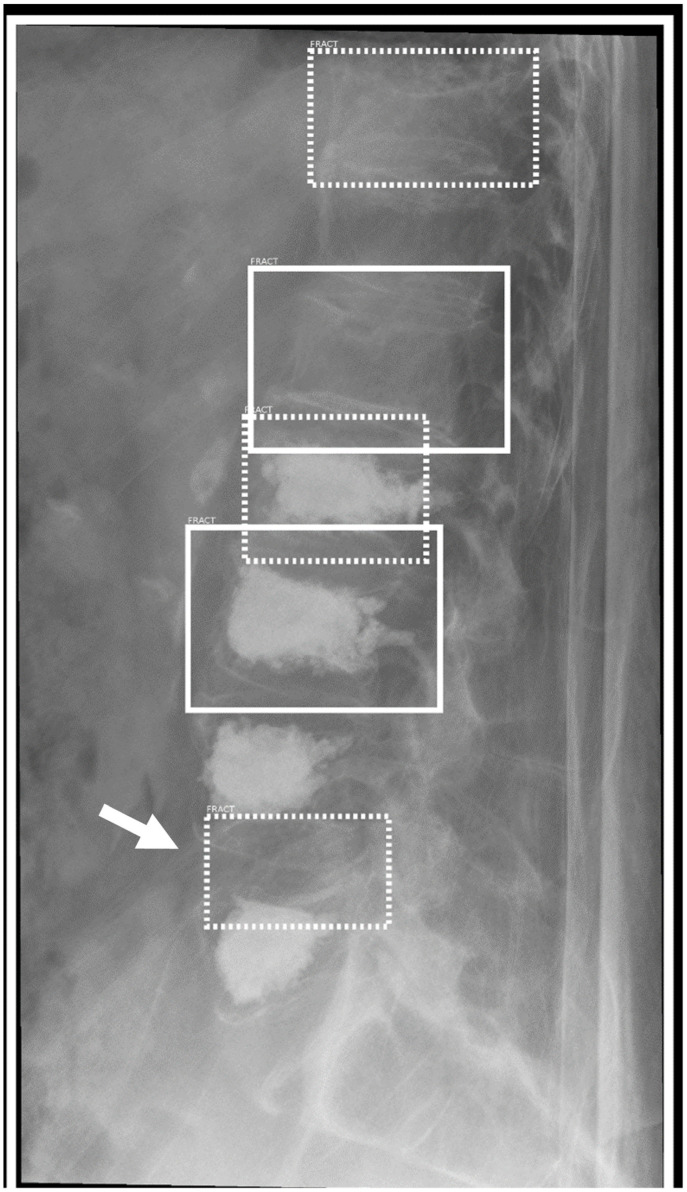
Lateral radiograph of the lumbar spine. Multiple (older) vertebral compression fractures are correctly marked as “Positive” (through line bounding box) and “Doubt” (dashed line bounding box). The bottom bounding box marks the intervertebral space L4/5 (arrow) an obvious mistake of the AI.

**Figure 7:**
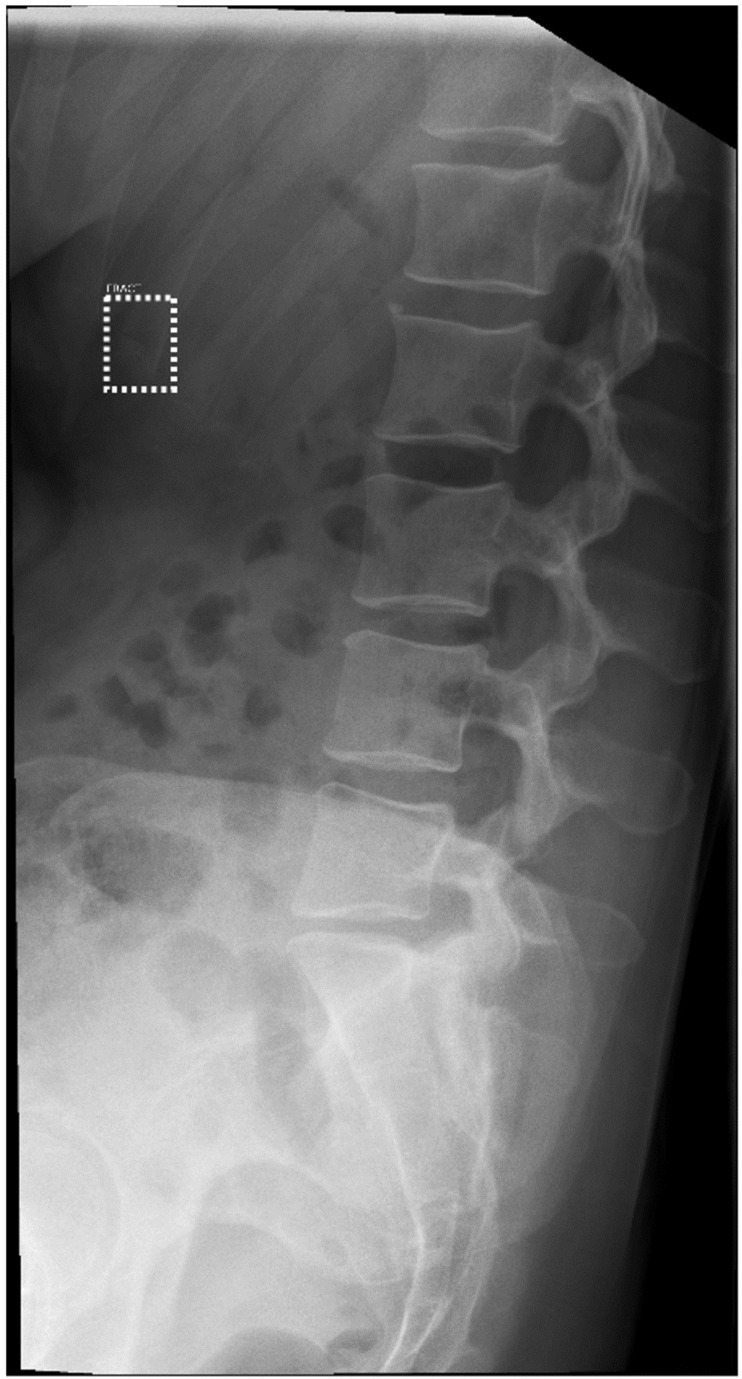
Lateral radiograph of the lumbar spine. In multiple such images, the AI incorrectly marks rib overlay as a possible fracture, noted here by the dashed bounding box marking a “Doubt” fracture.

The AI software offered a handful of instances with results that could best be described as “obvious mistakes”. In a few cases of lateral spine radiographs, intervertebral spaces were marked as a fracture (see Figure 6), possibly due to degenerative changes mimicking a fracture line. In other instances, rib overlay in radiographs was marked as a fracture (see Figure 7). These mistakes call into question the applicability of using an AI in a standalone setting, as they may result in an overcalling of fractures.

There are several possible limitations to our study. A limited number of residents participated within regular working hours in our research. Results may vary with a larger group of individuals depending on internal and external factors, such as prior experience. Results during night and weekend shifts may change reader performance. Gold standard in this study was set by the final report of the board-certified radiologist, which also represents a real-life approach. Here a limited number of wrong diagnoses may be present in the dataset. As the AI results are directly shown in our clinicals PACS system, the board-certified radiologist was able to see the AI results while correcting the initial report, an influence through this cannot be excluded. The influence on patient outcome of an increased accuracy in the preliminary report was not studied; further research is needed to determine if this leads to a reduction in additional imaging and/or better therapy. 

## 5. Conclusions

AI assistance in fracture detection was able to significantly increase the sensitivity for fractures by almost 7% (84.7 vs. 91.3%) in radiographs for resident radiologists without a loss of specificity (97.1% vs. 97.4%). Standalone AI performance was only slightly above human performance in terms of sensitivity at 86.9%, with a much lower specificity (84.7%), highlighting the advantages of a combined approach. The higher accuracy may reduce workload for board-certified radiologists in correcting preliminary reports and may lead to improvements in patient care through better initial diagnosis.

## Figures and Tables

**Table 1:** Full results for the full dataset, as well as each reviewer (±95% confidence interval).

		Sensitivity	Specificity	Positive Predictive Value	Negative Predictive Value
**Full set**	Human	84.74% (±0.4)	97.11% (±0.01)	93.11% (±0.03)	93.24% (±0.02)
	AI only	86.92% (±0.03)	84.67% (±0.03)	72.33% (±0.04)	93.35% (±0.02)
	Combined	91.28% (±0.03)	97.36% (±0.01)	94.10% (±0.02)	96.03% (±0.01)
**Reviewer 1**	Human	85.85% (±0.05)	97.29% (±0.01)	93.41% (±0.03)	93.90% (±0.02)
	AI only	84.34% (±0.05)	84.49% (±0.03)	76.61% (±0.06)	92.67% (±0.02)
	Combined	90.91% (±0.04)	97.98% (±0.01)	95.24% (±0.03)	96.02 (±0.02)
**Reviewer 2**	Human	83.43% (±0.06)	96.88% (±0.02)	92.76% (±0.04)	92.43% (±0.03)
	AI only	89.94% (±0.05)	79.89% (±0.04)	68.16% (±0.06)	93.31% (±0.03)
	Combined	91.71% (±0.04)	96.60% (±0.02)	92.81% (±0.04)	96.06% (±0.02)

**Table 2:** Sensitivity and specificity by anatomic region for human only readers, AI-only performance and combined results.

	Human Only		AI Only		Combined	
Region	Sensitivity	Specificity	Sensitivity	Sensitivity	Sensitivity	Sensitivity
Spine	92.39%	98.43%	89.13%	62.20%	94.57%	100.00%
Ribs	64.29%	91.89%	78.57%	72.97%	78.57%	91.89%
Shoulder/clavicle	88.89%	96.88%	91.11%	84.38%	93.33%	96.88%
Elbow/arm	76.00%	96.55%	80.00%	89.66%	88.00%	96.55%
Wrist/hand	78.26%	96.06%	86.96%	89.76%	95.65%	95.28%
Hip/pelvis	93.22%	99.79%	88.13%	89.76%	93.22%	98.79%
Knee/leg	86.96%	97.74%	86.96%	93.98%	91.30%	98.50%
Ankle/foot	82.86%	95.58%	88.57%	88.50%	88.57%	95.58%

## Data Availability

Any data presented in this study can be made available in a fully anonymized manner upon request.

## References

[1] Benjamens S., Dhunnoo P., Mesko B. (2020). The State of Artificial Intelligence-based FDA-Approved Medical Devices and Algorithms: An Online Database. NPJ Digit. Med..

[2] Artificial Intelligence and Machine Learning (AI/ML)-Enabled Medical Devices. https://www.fda.gov/medical-devices/software-medical-device-samd/artificial-intelligence-and-machine-learning-aiml-enabled-medical-devices.

[3] van Leeuwen K.G., Schalekamp S., Rutten M., van Ginneken B., de Rooij M. (2021). Artificial Intelligence in Radiology: 100 Commercially Available Products and their Scientific Evidence. Eur. Radiol..

[4] Guermazi A., Tannoury C., Kompel A.J., Murakami A.M., Ducarouge A., Gillibert A., Li X., Tournier A., Lahoud Y., Jarraya M. (2021). Improving Radiographic Fracture Recognition Performance and Efficiency Using Artificial Intelligence. Radiology.

[5] Duron L., Ducarouge A., Gillibert A., Laine J., Allouche C., Cherel N., Zhang Z., Nitche N., Lacave E., Pourchot A. (2021). Assessment of an AI Aid in Detection of Adult Appendicular Skeletal Fractures by Emergency Physicians and Radiologists: A Multicenter Cross-sectional Diagnostic Study. Radiology.

[6] Wei C.J., Tsai W.C., Tiu C.M., Wu H.T., Chiou H.J., Chang C.Y. (2006). Systematic Analysis of Missed Extremity Fractures in Emergency Radiology. Acta Radiol..

[7] Hallas P., Ellingsen T. (2006). Errors in Fracture Diagnoses in the Emergency Department--Characteristics of Patients and Diurnal Variation. BMC Emerg. Med..

[8] Pinto A., Berritto D., Russo A., Riccitiello F., Caruso M., Belfiore M.P., Papapietro V.R., Carotti M., Pinto F., Giovagnoni A. (2018). Traumatic Fractures in Adults: Missed Diagnosis on Plain Radiographs in the Emergency Department. Acta Biomed..

[9] Wood G., Knapp K.M., Rock B., Cousens C., Roobottom C., Wilson M.R. (2013). Visual Expertise in Detecting and Diagnosing Skeletal Fractures. Skeletal Radiol..

[10] Whang J.S., Baker S.R., Patel R., Luk L., Castro A. (2013). The Causes of Medical Malpractice Suits Against Radiologists in the United States. Radiology.

[11] Hayashi D., Kompel A.J., Ventre J., Ducarouge A., Nguyen T., Regnard N.E., Guermazi A. (2022). Automated Detection of Acute Appendicular Skeletal Fractures in Pediatric Patients using Deep Learning. Skeletal Radiol..

[12] Nguyen T., Maarek R., Hermann A.L., Kammoun A., Marchi A., Khelifi-Touhami M.R., Collin M., Jaillard A., Kompel A.J., Hayashi D. (2022). Assessment of an Artificial Intelligence Aid for the Detection of Appendicular Skeletal Fractures in Children and Young Adults by Senior and Junior Radiologists. Pediatr Radiol..

[13] Kuo R.Y.L., Harrison C., Curran T.A., Jones B., Freethy A., Cussons D., Stewart M., Collins G.S., Furniss D. (2022). Artificial Intelligence in Fracture Detection: A Systematic Review and Meta-Analysis. Radiology.

[14] Bluthgen C., Becker A.S., Vittoria de Martini I., Meier A., Martini K., Frauenfelder T. (2020). Detection and Localization of Distal Radius Fractures: Deep Learning System Versus Radiologists. Eur. J. Radiol..

[15] Lindsey R., Daluiski A., Chopra S., Lachapelle A., Mozer M., Sicular S., Hanel D., Gardner M., Gupta A., Hotchkiss R. (2018). Deep Neural Network Improves Fracture Detection by Clinicians. Proc. Natl. Acad. Sci. USA.

[16] Cheng C.T., Wang Y., Chen H.W., Hsiao P.M., Yeh C.N., Hsieh C.H., Miao S., Xiao J., Liao C.H., Lu L. (2021). A Scalable Physician-level Deep Learning Algorithm Detects Universal Trauma on Pelvic Radiographs. Nat. Commun..

